# Isolation of *Legionella pneumophila* by Co-culture with Local Ameba, Canada 

**DOI:** 10.3201/eid2511.190522

**Published:** 2019-11

**Authors:** Rafik Dey, Harley Mount, Alex W. Ensminger, Greg J. Tyrrell, Linda P. Ward, Nicholas J. Ashbolt

**Affiliations:** University of Alberta, Edmonton, Alberta, Canada (R. Dey, G.J. Tyrrell, N.J. Ashbolt);; University of Toronto, Toronto, Ontario, Canada (H. Mount, A.W. Ensminger);; Alberta Health Services, Edmonton (G.J. Tyrrell, L.P. Ward, N.J. Ashbolt)

**Keywords:** Legionellosis, case report, Legionnaires’ disease, hot tub, *Legionella pneumophila*, coculture, ameba, bacteria, Canada

## Abstract

Legionellosis was diagnosed in an immunocompromised 3-year-old girl in Canada. We traced the source of the bacterium through co-culture with an ameba collected from a hot tub in her home. We identified *Legionella pneumophila* serogroup 6, sequence type 185, and used whole-genome sequencing to confirm the environmental and clinical isolates were of common origin.

*Legionella pneumophila* is a waterborne bacterium responsible for Legionnaires’ disease, a potentially fatal respiratory disease acquired through environmental exposure to aerosolized water. According to the World Health Organization, *L. pneumophila* is the most common cause of legionellosis worldwide (*1*). For unknown reasons, cases reported in the United States and Europe have risen sharply over the past decade (*2*,*3*). Most legionellosis cases identified are caused by *L. pneumophila* serogroup 1 (*4*), possibly because of extensive use of initial urinary antibody screening that focuses on serogroup 1. 

Free-living amebae are known natural environmental reservoirs for *L. pneumophil*a (*5*). Amebae particularly play a role in legionellae growth in warm and stagnant engineered environments at 35°–45°C and in the bacterium’s persistence in high temperatures, biocides, and pH extremes (*6*). Co-culture with amebae is an efficient tool to detect *L. pneumophila* from human and environmental samples (*7*). However, amebae are rarely used in environmental investigations.

## The Study

In December 2016, an immunocompromised 3-year-old girl was admitted to Alberta Children’s Hospital in Calgary, Alberta, Canada, with acute respiratory distress syndrome and septic shock requiring extracorporeal membrane oxygenation. She was transferred to a pediatric intensive care unit in Edmonton, Alberta, Canada, where examination of the lungs confirmed pneumonia in the left lower lobe segment with a lung abscess. Empiric treatment for infections was started immediately with meropenem, vancomycin, tobramycin, azithromycin, and trimethoprim/sulfamethoxazole. Subsequent bronchoalveolar lavage and bronchoscopy were performed, along with microbial culture for identification of fungi, mycobacteria, mycoplasma, viruses, and *Legionella*. Public Health Laboratory (ProvLab), Edmonton, successfully isolated *Legionella* sp. from clinical samples by using buffered charcoal yeast extract (BCYE) medium, with and without antimicrobial drugs, including polymyxin B, cycloheximide, and vancomycin. The clinical isolate was identified as *L. pneumophila* by using *Legionella pneumophila* Direct DFA Kit (Pro-Lab Diagnostics, https://pro-lab.com) direct fluorescent antibody assay. The National Microbiology Laboratory in Winnipeg, Manitoba, typed the isolate as serogroup 6, sequence type 185 (ST185), confirming Legionnaires’ disease. 

The patient was treated with levofloxacin and a prophylactic dose of trimethoprim/sulfamethoxazole. She also received vancomycin, meropenem, and tobramycin for 8 days and azithromycin for 5 days. Her condition steadily improved, and she was discharged from the hospital a few days after treatment.

To investigate possible *L. pneumophila* sources, the Infection Prevention Control Research Laboratory of Alberta Health Services collected several first-flush water samples from sinks, a shower head, and a hot tub at the patient’s house and from sinks in the admitting hospital. All samples were negative for *L. pneumophila* by culture, but quantitative PCR results indicated the home hot tub was the likely source of the bacterium. 

We initially attempted co-culture with *Acanthamoeba polyphaga* (ATCC30461) (*8*) but failed to isolate *Legionella* spp. Because growth of *L. pneumophila* in the environment is hypothesized to be dependent partly on the composition of local amebic populations (*9*), we isolated free-living amebae hosts from the hot tub samples. We then identified ameba-resisting bacteria (ARB) by using the isolated ameba in co-culture with the hot tub samples (*7*).

**Figure 1:**
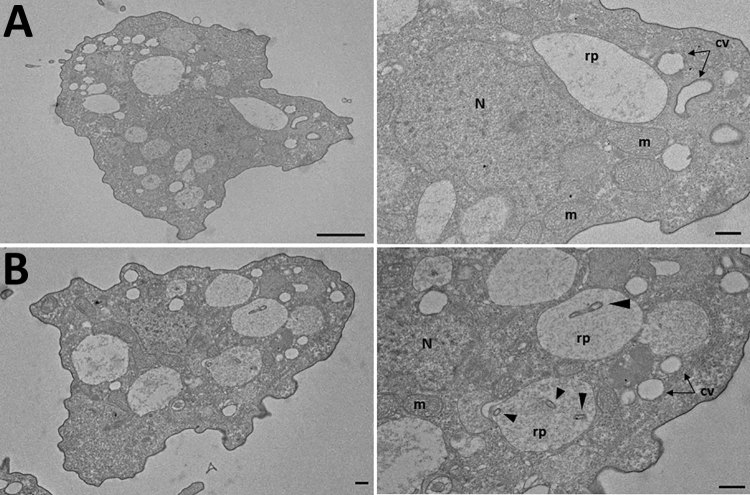
Transmission electron micrograph of amebae isolated from the home hot tub of a an immunocompromised 3-year-old girl with legionellosis before and after co-culture with *Legionella pneumophila*, Calgary, Alberta, Canada. A) Trophozoites of *Vermamoeba vermiformis* before co-culture. Note the absence of intracellular bacteria in the replicative phagosome. B) *V. vermiformis* replicative phagosome containing *L. pneumophila* serogroup 6 after 6 h of co-culture. Arrows indicate *L. pneumophila* contained within replicative phagosomes. Scale bars in left panels indicate 2 μm; scale bars in right panels indicate 500 nm. cv, contractile vacuoles; m, mitochondria; N, nucleus; rp, replicative phagosome.

We isolated free-living ameba hosts by filtering water from different environmental sites. We grew amebae at 30°C on nonnutrient agar supplemented with a thin film of viable *Escherichia coli* (ATCC25922). We isolated 2 free-living amebae, an *Acanthamoeba* sp. and a *Vermamoeba vermiformis*, and identified species by morphology (Figure 1, panel A) and 18S rRNA gene sequencing.

For co-culture experiments, we established amebae in axenic cultures in Nunc 25-cm^2^ tissue culture flasks (ThermoFisher Scientific, https://www.thermofisher.com) containing 5 mL serum casein glucose yeast extract medium at 37°C with 10% fetal calf serum. Before experiments, we performed subcultures of amebae every 3–4 days to ensure that trophozoites were in an exponential growth phase.

In brief, we co-cultured each environmental water sample with its isolated ameba by using several dilutions and incubating samples at 30°C for 12 h. When we observed amebal lysis, we recovered ARB on BCYE agar. We identified 1 of the ARB isolates from the ameba–hot tub culture as *L. pneumophila* by using 16S rRNA gene sequencing (Table) and subsequent sequence-based typing (*10*). Serotyping for *L. pneumophila* indicated both the clinical and environmental isolates were ST185, serogroup 6. We confirmed the presence of *L. pneumophila* inside *V. vermiformis* replicative phagosomes by transmission electron micrograph (Figure 1, panel B).

**Table:** Bacteria isolated from water samples by co-culture with local ameba and location of water samples in investigation of a legionellosis case, Calgary, Alberta, Canada

Ameba host and bacterium	Water sample location
*Acanthamoeba* sp.	
* Pseudomonas stutzeri*	Hospital sink
* Paenibacillus terrigena*	Hospital sink
* Pseudacidovorax intermedius*	Hospital sink
*Vermamoeba vermiformis*	
* Acidovorax delafieldii*	Home hot tub
Legionella pneumophila*	Home hot tub
*L. pneumophila isolate from the case-patient’s home hot tub was confirmed as the same serotype and sequence type as the clinical isolate from the case-patient.	

For confirmation, we performed whole-genome sequencing on clinical and environmental isolates. We extracted genomic DNA by using the NucleoSpin Tissue Kit (Macherey-Nagel, https://www.mn-net.com). We prepared libraries according to the protocol for the Nextera XT DNA Library Prep Kit (Illumina, https://www.illumina.com) and sequenced on an Illumina MiniSeq by using 2 × 150-nt reads. We deposited sequence information into BioProject (https://www.ncbi.nlm.nih.gov/bioproject) under accession no. PRJNA482644. 

We trimmed sequence reads by using Trimmomatic version 0.36 (*11*) with the following parameters: Nextera clip, 2:30:10:8:true; LEADING, 20; TRAILING, 20; SLIDINGWINDOW, 4:20; MINLEN, 36. We assembled reads by using the SPAdes version 3.12 assembler in careful mode (*12*). We identified the closest related *L. pneumophila* strains for each set of contigs by using the PATRIC server (*13*) and searching for whole genome k-mer. We reordered assembled contigs by using mauve (*14*) against the closest matching reference, ATCC43290. We mapped trimmed sequence reads from both strains to the clinical isolate in the SPAdes de novo assembly by using Bowtie2 version 2.3.4.1 (https://github.com/BenLangmead/bowtie2), then processed reads with Picard (Broad Institute, https://www.broadinstitute.org) and identified single-nucleotide polymorphism insertions/deletions by using FreeBayes version 1.2.0 (https://www.geneious.com/plugins/freebayes). We filtered resulting polymorphisms with VCF.Filter version 4.2 (https://biomedical-sequencing.at/VCFFilter) by using QUAL >10 & DP >20 & QUAL/AO >10 & SAF >0 & SAR >0 & RPR >1 & RPL >1. To rule out assembly errors or other spurious calls, we visually inspected the location of each putative polymorphism in reference assemblies of each isolate and traced back to the clinical contigs. 

We constructed a phylogenetic tree by submitting assembled scaffolds to the RAST server for genome annotation (*15*). To find core conserved orthologs with default parameters, we input the resulting gene annotations into OrthoMCL (https://orthomcl.org), alongside the complete ORFeomes of 6 other *L. pneumophila* strains: Philadelphia-1 NC_002942.5, Lens NC_006369.1, Thunder Bay CP003730.1, 570-CO-H NC_016811.1, Toronto-2005 NZ_CP012019.1, and Calgary-2012 SAMN03944918. We individually aligned 2,403 identified orthologs (2,471,034 nt) across all strains by using the MUSCLE algorithm (https://www.ebi.ac.uk/Tools/msa/muscle) and concatenated orthologs into a superalignment for tree construction. We adopted RAxML version 8.2.12 (ILRI Research Computing, http://hpc.ilri.cgiar.org) with a general time-reversible nucleotide substitution model for 1,000 bootstraps to generate a maximum-likelihood phylogenetic tree.

**Figure 2:**
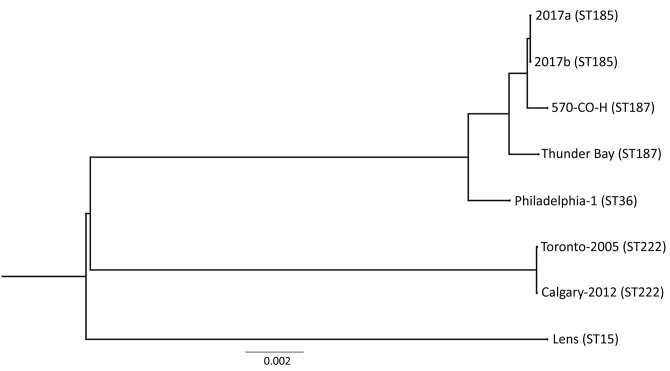
Phylogenetic tree depicting the relationship between *Legionella pneumophila* isolates identified during investigation of legionellosis in an immunocompromised 3-year-old girl, Calgary, Alberta, Canada, and reference sequences. *L. pneumophila* core ortholog-based maximum-likelihood phylogenetic tree shows 8 previously published genomes and sequences of the 2 isolates from this study (2017a, clinical isolate from patient; 2017b, environmental isolate from hot tub in patient’s home). Tree construction was performed by using 2,403 orthologous sequences (2,471,034 nt). Each ortholog sequence was independently aligned with the MUSCLE algorithm (https://www.ebi.ac.uk/Tools/msa/muscle) and concatenated into a single superalignment, which then was subjected to 1,000 bootstrap iterations to find the maximum-likelihood phylogeny. Scale bar indicates the number of nucleotide substitutions per site. ST, sequence type.

Results of whole-genome sequencing analysis strongly suggest that clinical isolate 2017a and environmental isolate 2017b from the patient’s home hot tub were of common origin. With only a few single-nucleotide polymorphism differences (Figure 2), these data indicate the hot tub was the source of the patient’s infection.

Of note, we were not able to recover any legionellae from environmental samples with initial BCYE and other direct culture approaches. *L. pneumophila* serogroup 6 has not been identified in previous hot tub–associated infections, and this case might have been a result of the patient’s immunocompromised status. A commercial hot tub cleaning product was subsequently used for disinfection and maintenance of the hot tub. In a 1-year follow up analysis, water samples from the hot tub were negative for amebae and any *Legionella* spp.

## Conclusions

Our report demonstrates the utility of ameba co-culture and emphasizes the use of locally sourced ameba to recover the source of *L. pneumophila* from environmental samples. Our findings also suggest that investigations should include free-living ameba to indicate the presence of potentially pathogenic *Legionella* spp. and as a potential factor to minimize the need for remediation actions associated with contaminated environments.
